# The amygdala between sensation and affect: a role in pain

**DOI:** 10.1186/2049-9256-1-9

**Published:** 2013-06-05

**Authors:** Pierre Veinante, Ipek Yalcin, Michel Barrot

**Affiliations:** Institut des Neurosciences Cellulaires et Intégratives, UPR3212, Centre National de la Recherche Scientifique, 21 Rue René Descartes, 67084 Strasbourg Cedex, France; Université de Strasbourg, 21 Rue René Descartes, 67084 Strasbourg Cedex, France

**Keywords:** Amygdala, Hypoalgesia, Hyperalgesia, Affective/emotional component, Persistent pain

## Abstract

The amygdala is a structure of the temporal lobe thought to be involved in assigning emotional significance to environmental information and triggering adapted physiological, behavioral and affective responses. A large body of literature in animals and human implicates the amygdala in fear. Pain having a strong affective and emotional dimension, the amygdala, especially its central nucleus (CeA), has also emerged in the last twenty years as key element of the pain matrix. The CeA receives multiple nociceptive information from the brainstem, as well as highly processed polymodal information from the thalamus and the cerebral cortex. It also possesses the connections that allow influencing most of the descending pain control systems as well as higher centers involved in emotional, affective and cognitive functions. Preclinical studies indicate that the integration of nociceptive inputs in the CeA only marginally contributes to sensory-discriminative components of pain, but rather contributes to associated behavior and affective responses. The CeA doesn’t have a major influence on responses to acute nociception in basal condition, but it induces hypoalgesia during aversive situation, such as stress or fear. On the contrary, during persistent pain states (inflammatory, visceral, neuropathic), a long-lasting functional plasticity of CeA activity contributes to an enhancement of the pain experience, including hyperalgesia, aversive behavioral reactions and affective anxiety-like states.

## Background

Many cerebral structures, constituting the pain matrix, participate in nociception and in the expression and modulation of pain. They transmit and decode nociceptive information, generate, amplify or reduce the pain sensation, allow expression of defensive or distress behavior, and influence the affect. Among these brain structures, the amygdaloid complex, or amygdala, appears as a key component of the pain matrix [1]. Indeed, while this group of sub-cortical nuclei within the temporal lobe is well known for its critical role in the control of emotions [2], it has also been the target for a large number of fundamental research in the pain field during the two past decades. Overall, the amygdala is considered to provide an emotional value – either positive or negative – to sensory information, thus leading to adapted behavioral and affective responses and contributing to emotional memory. This role has been more particularly studied in the conditioned fear experimental paradigm [2–4]. Such conditioning is an adaptive process in response to potential danger. As nociceptive stimulations are also potentially harmful for body integrity, it is not surprising that the amygdala can integrate various nociceptive information to initiate or modulate the autonomic and behavioral responses according to the environmental context (internal and external) and to the affective state. This adaptation includes mechanisms of hypoalgesia/analgesia that allows reflex inhibition and facilitates fight or flight responses. Moreover, this brain structure participates in the anticipation/prediction of potential danger, based on salient sensory cues (sounds, images, odors…), to initiate the same adaptive responses as in direct presence of the danger. This is for example leading to conditioned or to stress-induced analgesia.

Pain includes a strong emotional component making it aversive and a reciprocal relation exists between pain and affective states. Stress can either inhibit or amplify pain; and anxiety as well as depressive states are often associated with more intense pain sensation, particularly when pain becomes chronic. In such chronic pain state, the amygdala could contribute to the hyperalgesia as well as to the anxio-depressive consequences of pain.

A few publications reviewed the role of the amygdala in persistent pain [5, 6]. The present review will consider the recent published data on the subject and address the key role of amygdala, especially its central nucleus, in various pain processes, either physiological or pathological. This will be done by reminding the anatomical context and presenting the morphological and functional evidences allowing insertion of the amygdala within the pain matrix. Then, we will show that the amygdala can exert either inhibitory or facilitating action on nociception and pain as well as on its affective component and consequences. Finally, we will address recent electrophysiological, neurochemical and biochemical data that are giving insights into underlying cellular and molecular mechanisms. While experimental data that are presented concern studies conducted in rodents, we will confront them with human brain imaging studies, thus stressing the potential role of the amygdala in human pain, especially with clinical relevance.

## Review

### Amygdala and extended amygdala

**Figure 1:**
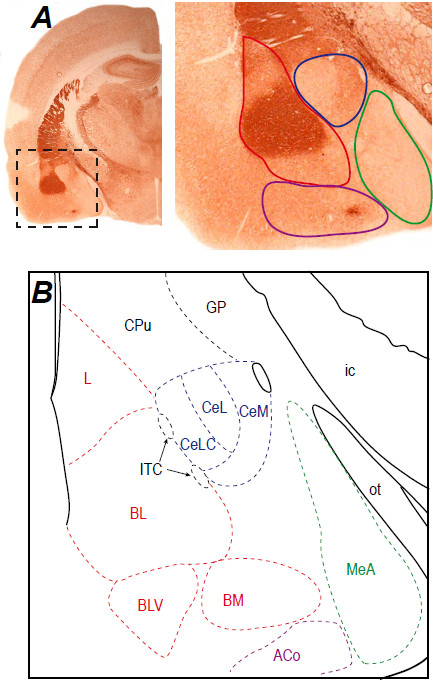
**Anatomical organization of the amygdala. A**: Frontal section of a rat brain at the level of the amygdala processed for acetylcholinesterase staining. The boxed area indicates the position of the magnified region right-hand. The four main groups are outlined: superficial (purple), basolateral (red), central (blue), medial (green). **B**: Schematic representation of the main amygdala nuclei. The basolateral group (red) includes the lateral (L), basolateral (BL), basolateral ventral (BLV) and basomedial (BM) nuclei. The central group is represented by the central nucleus with its capsular (CeLC), lateral (CeL) and medial (CeM) subdivisions. The medial (green) and superficial (purple) groups are represented at this level by the medial (MeA) and anterior cortical (ACo) nuclei, respectively. The intercalated cell masses (ITC, black) appear wedged between BL and CeA. Other abbreviations: CPu: caudate-putamen; ic: internal capsule; GP: globus pallidus; ot: optic tract. Photographs of acetylcholinesterase staining from the authors’ archive.

The amygdala is a nuclear complex with almond shape, located in the temporal lobe of mammals and identified by Burdach at the start of 19th century. It is an heterogeneous structure, grouping around a dozen of nuclei depending on the considered nomenclature, these nuclei being classically clustered in 4 groups: superficial, basolateral, central and medial (Figure 1) [7, 8]. The first two groups constitute the corticobasal amygdala that shows cortical-like characteristics [8]. Its main nuclei, the lateral and the basolateral nuclei are known as the basolateral amygdala (BLA) and mostly contain projection neurons of pyramidal type, synthesizing glutamate. It is reciprocally connected with the cerebral cortex, and sends a major projection to the striatal complex, especially in its ventral part [9, 10]. The BLA also densely innervates the central and medial groups of amygdala nuclei. These last two groups display an organization that is similar to the one of the basal ganglia, with striatopallidal-like neuronal morphology [8, 11]. Moreover, the central and the medial amygdala groups receive information from the cerebral cortex but do not send any direct projection to it. In addition to the four groups, a few nuclei of the amygdala remain unclassified, among them the intercalated cell masses which are small clusters of densely packed GABAergic neurons [12].

The central nucleus of the amygdala (CeA), main component of the central group of amygdala nuclei, is considered as the output nuclei of the amygdaloid complex. It integrates information treated by the corticobasal group and it influences effector centers. The CeA is also reciprocally connected to more rostral forebrain structures, such as the lateral part of the bed nucleus of the stria terminalis (BSTL) and the dorsal part of the substantia innominata. This group of interconnected structures presents strong morphofunctional homogeneity and is designed as the central extended amygdala [8, 13]. In this review, we will more specifically detail data on the CeA, which is the most studied amygdaloid structure for its role in nociception and pain, and also refer to findings on the extended amygdala, as CeA functions are closely related to this forebrain macrostructure.

**Figure 2:**
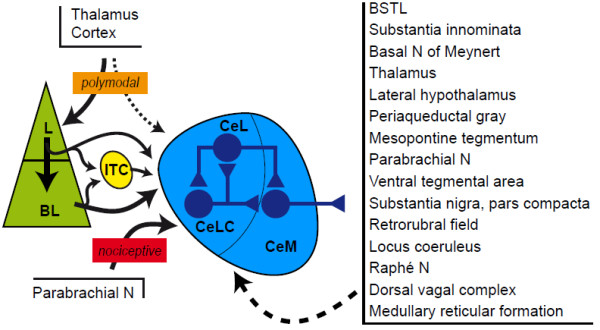
**Main connections of the CeA involved in pain processes.** The CeA received highly processed polymodal information from the cerebral cortex and the thalamus essentially through inputs from the lateral (L) and basolateral (BL) nuclei, and more direct nociceptive information from the parabrachial nucleus. Intercalated cell masses (ITC) provide an inhibitory input driven by L and BL. Most of these afferents contact neurons in the capsular (CeLC) and lateral (CeL) subdivisions of the central nucleus which control the activity of neurons in the medial subdivision (CeM). Efferents arise mainly from the CeM and target the other components of the central extended amygdala, thalamic nuclei and the integrative, modulator and effector centers in the hypothalamus and the brainstem. Most of these structures send a reciprocal projection to the central nucleus.

The CeA has three subdivisions: capsular (CeLC), central (CeL) and medial (CeM), that are defined based on cytoarchitecture, neurochemistry and connectivity [8, 11]. The CeLC and the CeL mostly contain medium-size spiny striatal-like neurons, while the CeM main neuronal population has a leptodendritic pallidal-like morphology (i.e. neurons with long dendrites with few or no spines). Neurons from these 3 sub-divisions co-synthesize GABA and neuropeptides, such as opioid peptides (enkephalins, dynorphin, endorphins), corticotropin-releasing factor (CRF), neurotensin, somatostatin, galanin or substance P [11, 14–16]. These peptides and others, such as calcitonin gene-related peptide (CGRP), are also present in a dense neuropile of incoming axonal terminals [11]. Connections of the CeA (Figure 2) are characterized by strong relation, often reciprocal, with autonomic and modulatory centers of the hypothalamus and the brainstem. This includes the dorsal vagal complex, the parabrachial nucleus, the periaqueductal gray (PAG), the medullary and mesopontine reticular formation, the paraventricular nucleus of the hypothalamus and the lateral hypothalamus [7, 8, 11]. The midbrain dopaminergic centers, the locus coeruleus and the dorsal raphe are also under CeA control, and the CeA receives monoaminergic inputs from these structures. The connections of the CeA with the thalamus are sparser but reciprocal, and concern the paraventricular, posterior intralaminar and subparafascicular nuclei [8]. Cortical inputs mainly arise from non-isocortical areas (prefrontal, insular and perirhinal cortices) and from the corticobasal nuclei of the amygdala [11, 17]. While the CeA doesn’t directly project to the cerebral cortex, it has outputs toward substantia innominata cholinergic neurons that are projecting to the cortex [8]. The amygdaloid intercalated cell masses also provide an intra-amygdaloid input to the CeA [12]. Last, the CeA is interconnected with other components of the central extended amygdala through a dense network of reciprocal projections. Within the CeA, information follow a latero-median flux [11]: most afferents do synapses in the CeLC and the CeL, and neurons of these two subdivisions then influence the CeM where most of the efferent projection neurons of the CeA are found (Figure 2). Globally, these morphofunctional characteristics are similar to other components of the central extended amygdala [8]. This general organization of amygdala and extended amygdala described in rodent is similar to the human brain [8, 13].

### The CeA receives multiple nociceptive informations

Among the various CeA afferents, two main pathways are preferentially conveying nociceptive information (Figure 2). A first pathway originates from the BLA, and conveys highly integrated polymodal information, including nociceptive, from the thalamus and the cerebral cortex, allowing for example fear conditioning [2, 4, 7]. A cascade of projections, originating in the ventroposterior, posterior, triangular and posterior intralaminar nuclei of the thalamus, the second somatosensory area and the insular cortex, brings nociceptive information to the BLA which in turn transmits it to the CeA [7, 17, 18]. More precisely, the lateral nucleus of the amygdala projects lightly to the CeLC and the CeL and massively to the basolateral nucleus which targets preferentially to the CeM [4, 7, 11]. Additionally, lateral and basolateral projections to the CeA can be relayed in the intercalated cell masses, providing an inhibitory interface in the BLA-CeA pathway [4, 12]. Moreover, the CeA also receives less dense but direct projection from these thalamic and cortical areas [17, 18]. These largely polysynaptic efferents thus allow the CeA to integrate a nociceptive information which already acquired affective and cognitive significance in cortical circuits.

A second pathway conveys to the CeA more direct and raw nociceptive information. While spinal cord only sparsely sends direct projection to the amygdala, spinal nociceptive information is largely transmitted to the CeA via the parabrachial nucleus (PB). This pontine integrative center is a major target for superficial layers of the spinal cord, but also for deeper layers, for the trigeminal complex and for the nucleus of the solitary tract [19, 20]. The nociceptive aspect of these afferents was clearly established by electrophysiology [20]. The PB thus gathers nociceptive information from all cutaneous, deep tissue and visceral territories. Three main ascending pathways are then originating from the PB, toward the medial thalamus, the medial hypothalamus and the central extended amygdala [20]. The parabrachio-amygdaloid pathway is highly organized, with a specific topography connecting various PB subdivisions to the ones of the CeA and the BSTL [19, 20]. Moreover, unitary analysis of parabrachio-amygdaloid axonal branching showed that the different components of the central extended amygdala are innervated by axonal collaterals from a same PB neuronal population that never send projection to the medial thalamus or the medial hypothalamus [21].

*In vivo* electrophysiological studies allowed characterizing the response of CeA neurons to nociceptive stimulations [6, 19, 20, 22, 23]. In anesthetized rats, cutaneous (mechanical or thermal), deep tissue (articular and muscular) and visceral nociceptive stimulations provoke changes in the activity of a large majority of CeA neurons (Table 1). Most of them, particularly in the CeLC, are activated by nociceptive stimuli, either exclusively or preferentially, while fewer neuronal inhibition can be observed in the CeL. Few CeM neurons are responding to nociceptive stimuli. This has led to define the CeLC as the “nociceptive amygdala” [6] even though contributions from the CeL to nociceptive process cannot be ruled out. The CeA neurons can encode the stimulus intensity, as shown by sigmoid stimulus–response curves. Receptor fields are particularly large, often bilateral and can cover the whole body [20, 22, 23]. These neurons often respond to both cutaneous and deep tissue stimulation, whether mechanical or thermal. Together, these data suggest that even though CeA neurons can detect nociceptive information, the stimulus–response shape of this response as well as the wide receptor fields do not allow the CeA to play a major role in the sensori-discriminative aspect of pain. This brain region rather contributes to pain controls and to the emotional and affective components of pain [5, 6, 19, 20].

**Table 1:** **Reported changes in the rodent CeA in different pain situations**

Pain situation^a^	Changes in the CeA	Reference
*Acute somatic stimulations*	Changes (mostly excitation) in electrical activity	[22, 23]
*Visceral pain*		
ip acetic acid	- Increased *c-fos* mRNA expression	[24]
Esophageal acetic acid	- Increased c-Fos immunoreactivity	[25]
Colitis	- Increased neuronal excitability	[26, 27]
	- Enhanced the PB-CeA, but not the BLA-CeA transmission	
	- Increased *crf* mRNA expression	
Cystitis	- Increased c-Fos and Krox-24 imunoreactivities	[28, 29]
	- Increased *crf* mRNA expression	
*Inflammatory pain*		
Intraplantar formalin	- Induced ERK activation in the right CeA	[30, 31]
Acid-induced muscle pain	- Increased ERK activation	[32]
	- Enhanced the PB-CeA transmission	
Knee joint arthritis	- Increased spontaneous activity in the right CeA	[33–36]
	- Increased neuronal excitability in the right CeA	
	- Enhanced the PB-CeA and the BLA-CeA transmission	
	- Increased mGluR1 and mGluR5 expression	
	- Increased phosphorylation of NR1 subunit	
*Neuropathic pain*		
Sciatic nerve ligation or section	- Increased spontaneous and evoked activity differentially in the left and right CeA	[37–41]
	- Enhanced the PB-CeA transmission	
	- Increased *crf* mRNA expression and CRF immunoreactivity	
	- Increased glucocorticoid receptor mRNA expression	
	- Increased cell proliferation	

^a^ Pain situation: acute somatic stimulations: nociceptive mechanical pinches or thermal stimulations applied on different body parts; ip acetic acid: intraperitoneal injection of acetic acid; colitis: inflammation by intracolonic infusion TNBS (2,4,6-trinitrobenzenesulfonic acid) or zymosan; cystitis: urinary bladder inflammation by intraperitoneal injection of cyclophosphamide; intraplantar formalin: intraplantar injection of diluted formalin in hindpaw; Acid-induced muscle pain: intramuscular injection of acidic saline (pH4) in gastrocnemius; knee-joint arthritis: intraarticular injection of kaolin-caragenean in knee; Sciatic nerve ligation or section: loose or tight ligation of sciatic nerve or spinal roots, or sparing section of roots.

The recruitment of the CeA in visceral and inflammatory pain has also been evidenced using the expression of immediate early genes such as *cfos* (Table 1). For example, intraperitoneal or esophageal acetic acid injection [24, 25], colorectal distension [42] or experimental cystitis [28] induce cFos in the CeA and the BSTL. Interestingly, this cellular recruitment in the CeA is not observed after intradermal formalin injection in the hindpaw. In this classical inflammatory pain model, cFos recruitment rather appears in the basolateral nucleus of the amygdala [24]. However, extracellular signal-regulated kinase (ERK) phosphorylation is observed in the CeA after intraplantar formalin [30] or in acid-induced muscle pain [32]. Last, in visceral pain models but also in more persistent pain models, such as knee intraarticular injection of kaolin-carrageenan, or neuropathic models based on sciatic nerve ligation, major changes can be observed in the CeA (Table 1), such as an increase in spontaneous and evoked activity [33, 34, 37], an enhancement of synaptic transmission at the PB-CeA and the BLA-CeA synapses [35, 38], an increase in the phosphorylation of NR1 glutamatergic subunit [36], an increased expression of CRF, of group I metabotropic glutamatergic receptors (mGluRs) and of corticosterone receptor [26, 29, 35, 39, 40] and an increased cell proliferation [41].

Human brain imaging confirms a role of the amygdala in pain processes [1]. However, the spatial resolution only allows a crude discrimination of the various nuclei of the amygdaloid complex. Globally, while older studies failed to detect pain-related changes in the amygdala, more recent studies revealed unilateral and bilateral activation or inhibition. These changes were observed after nociceptive thermal laser stimulation [43, 44] or colorectal distention [45]. The amygdala response is usually correlated to the intensity of the nociceptive stimulus, however the importance of attention and emotional factors has also been evidenced. Indeed, repeated thermal nociceptive stimulations of increased intensity lead to an activation of the amygdala that is correlated to the pain perception [43]. However, when sub-threshold stimulus is interpreted as painful by the subject, this situation may activate the amygdala similar to a nociceptive painful stimulus reflecting a state of anxiety or the anticipation of a potentially aversive event. In another study [44], the subjects were informed that they were about to be expose to a cold painful stimulus lasting 1 minute, or 2 minutes. Functional imaging during the first minute of stimulation revealed that the anticipation of longer stimulus duration led to the deactivation of the amygdala. This suggests that the impact of nociceptive information on the amygdala depends on the context, and not simply of the intrinsic properties of the stimulus.

### The CeA influences the nociceptive centers

The CeA receives and integrates nociceptive information, but it also influences the main pain centers. The CeA massively projects to other components of the central extended amygdala, to the lateral hypothalamus and to the brainstem [8]. Anatomical data indicate that the intrinsic and the extrinsic projections to the central extended amygdala arise from distinct neuronal populations [11]. Indeed, studies of unitary axonal reconstruction [46] show that medium size spiny neurons of the CeLC and the CeL mostly project to the BSTL and the dorsal substantia innominata, while axons of CeM projection neurons innervate the various brainstem structure through collateral branches. Since the nociceptive inputs from the PB preferentially target the CeLC and the CeL, such organization suggest that nociceptive information are integrated through an interneuron network intrinsic to the CeA and to the central extended amygdala before being delivered to output neurons influencing effector centers of the brainstem. Some of these centers are part of the nociceptive descending controls. Partially opioidergic CeA outputs are for example innervating the PAG that is a key element of descending controls of nociception through its influence on the ventromedial reticular formation [47]. The PB is also part of descending controls and it receives dense inputs from the CeA [8]. Beside these projections toward integrative centers, the CeA also directly projects to the ventromedial reticular formation [48], the dorsal reticular nucleus [49] and monoaminergic centers such as the substantia nigra and the rostral ventral tegmental area (dopamine), the locus coeruleus (noradrenaline) and the raphe nuclei (serotonin) [48, 50–52]. These structures are known to influence the nociceptive message. Moreover, monoaminergic centers projecting to the forebrain modulate striatal and cortical activities, potentially influencing affective, emotional and motivational aspects of pain. Finally, within the central extended amygdala, the dorsal substantia innominata that receives strong inputs from the CeA – and particularly from the CeLC – includes cholinergic neurons that innervate the prefrontal and insular cortices [8, 20].

Thus, the CeA has connections allowing the modulation of both the sensory and the affective, emotional and cognitive aspects of pain.

### Amygdala, acute pain and analgesia

The potential role of the amygdala in the modulation of pain has been suggested for a long time. It has been clearly shown that the anti- and pro-nociceptive effects are dependent on (1) the type of pain (acute, inflammatory or chronic); (2) the measured parameters (threshold or latency of reflex withdrawal, vocalizations, emotional component); and (3) the emotional state of the subjects (stress, anxiety, fear and expectation).

Nociceptive tests, relying on the latency of appearance of avoidance behavior or on the stimulus threshold, are used to determine the analgesia or hyperalgesia [53]. These tests include for example the tail-flick (tail withdrawal after a thermal stimulus), flinch test (paw or tail withdrawal after electrical stimulation), hot-plate (paw licking or jump after thermal stimulation) or Randall-Selitto (paw withdrawal or vocalization after mechanical stimulation). In these tests, the bilateral lesion or chemical inactivation of the amygdala causes neither anti-nociceptive nor pro-nociceptive effects in naïve animals [54–56]. However, injections of neuropeptides such as oxytocin, vasopressin, neurotensin, galanin, CRF or CGRP [57–62], or of noradrenergic or cholinergic agonists [63–66] into the CeA induce antinociceptive effects (Table 2). In addition, the amygdala is involved in the analgesic action of systemically administered morphine and cannabinoids since the lesion or the inactivation of the CeA strongly reduces the morphine- or cannabinoid-induced analgesia as measured by the tail-flick test [56, 67]. A recent study also showed that deleting the gene of brain-derived neurotrophic factor (BDNF) in parabrachio-amygdaloid neurons decreased the analgesic properties of morphine in mechanical and thermal nociceptive modalities [68]. Finally, the antinociceptive properties of intra-CeA morphine or intra-CeA β-endorphin are dependent upon opioidergic transmission in the amygdala-PAG pathway [47].

**Table 2:** **Effects of CeA manipulation on pain-related outcomes in different pain models**

Pain type^a^	Pain related outcome^b^		Reference
	Nociceptive behavior	Affective/emotional	
*1. CeA lesion*			
Naïve	- Reduced morphine-induced, stress-induced and conditioned hypoalgesia		[55, 56, 69]
Formalin	- Reduced morphine-induced and conditioned hypoalgesia	- Decreased pain-induced CPA	[70–73]
Acetic acid		- Decreased pain-induced CPA	[72]
*2. Injection of muscimol*			
Neuropathy	- Reduced mechanical hyperalgesia	- Decreased escape/avoidance	[74]
*3. Injection of NMDA antagonist*			
Neuropathy		- Decreased pain-induced CPA	[75]
*4. Injection of group I mGluRs ligands*			
Naïve	- Agonist induced visceral and mechanical hypersensitivity		[76, 77]
	- Antagonist reduced visceral sensitivity		
Formalin	- Antagonist reduced mechanical hypersensitivity		[77]
Arthritis	- Antagonist reduced mechanical hypersensitivity	- Antagonist decreased vocalizations	[78]
Neuropathy		- Agonist increased, and antagonist decreased, pain-induced CPA	[75]
*5. Injection of group III mGluRs agonists*			
Naïve	- Decreased mechanical sensitivity (mGluR7)	- Decreased vocalizations and anxiety	[79]
Arthritis	- Increased mechanical sensitivity (mGluR8)	- Increased vocalizations and anxiety	[79]
*6. Injection of cholinergic agonists*			
Naïve	- Decreased thermal sensitivity, reduced jaw opening reflex	- Decreased vocalizations	[63, 65, 66]
*7. Injection of noradrenergic α*_*2*_ *ligands*			
Naïve	- Agonist induced mechanical and thermal hypoalgesia		[64, 80]
	- Antagonist reduced stress-induced thermal hypoalgesia		
Acetic acid		- Agonist decreased pain-induced CPA	[81]
*8. Injection of noradrenergic β antagonists*			
Acetic acid		- Decreased pain-induced CPA	[81]
*9. Injection of CGRP receptor ligands*			
Naïve	- CGRP decreased mechanical and thermal reflexes		[59]
Naïve	- CGRP increased mechanical reflexes	- CGRP increased vocalizations	[82]
Arthritis	- CGRP1 antagonist inhibited the enhanced reflex to mechanical stimulus	- CGRP1 antagonist decreased vocalizations	[83]
*10. Injection of CRF receptor ligands*			
Naïve	- CRF decreased mechanical and thermal sensitivity		[58]
Naïve	- CRF increases mechanical sensitivity	- CRF increased vocalizations	[84]
Arthritis	- CRF1 antagonist reduced mechanical hypersensitivity	- CRF1 antagonist decreased vocalizations and anxiety	[85, 86]
*11. Injection of oxytocin, vasopressin, neurotensin, galanin*			
Naïve	- Decreased mechanical and/or thermal sensitivity		[57, 60–62]
*12. Injection of opioid receptors ligands*			
Naïve	- Morphine and β-endorphin induced mechanical and thermal hypoalgesia	- Morphine decreased vocalizations	[47, 66]
*13. Corticosterone implants*			
Naïve	- Sensitized visceromotor reflexes to colorectal and urinary bladder distension and to somatic mechanical sensitivity	- Increased anxiety	[87–90]
*14. BDNF gene deletion in the PB-CeA pathway*			
Naïve	- Decreased morphine-induced mechanical and thermal hypoalgesia		[68]
*15. Intracellular effectors*			
Naïve	- ERK activator induced mechanical hypersensitivity		[30]
Formalin	- ERK activation inhibitor decreased mechanical hypersensitivity		[30, 31]
Arthritis	- ERK activation inhibitor and PKA inhibitor decreased mechanical hypersensitivity	- ERK activation inhibitor and PKA inhibitor decreased vocalizations	[91]

^a^Pain type. naive nociceptive testing on naive animals; formalin: intraplantar injection of diluted formalin in hindpaw; acetic: intraperitoneal injection of acetic acid; arthritis: intraarticular injection of kaolin-caragenean in knee; neuropathy: compression or ligation of sciatic nerve or spinal root.

^b^Pain related outcome. thermal sensitivity: latency of withdrawal or escape in tail-flick, hot-plate or Heargraves tests; mechanical sensitivity: latency or threshold to withdrawal in von Frey or Randall-Sellito tests; CPA: pain-induced conditioned place aversion; vocalizations: intensity/duration/threshold of vocalizations to electrical shock (naive) or mechanical compression of knee (naive, arthritis); anxiety: anxiety-like behavior in elevated plus maze test.

These data therefore show that even if the CeA is not directly involved in the modulation of basal nociceptive thresholds in normal situations, it strongly influences analgesia processes (Table 2).

Stressful situations (restraint or noise) and fear, especially the experimental model of fear conditioning, cause analgesia as measured by the tail flick or the hot-plate tests. Several studies demonstrated that bilateral lesion or inactivation of the CeA diminishes or abolishes this analgesic effect [55, 69, 70, 80, 92] (Table 2). In parallel, clinical studies showed that stressful stimuli, either painful (electric shocks) or painless (unexpected noises), and innocuous stimuli, previously associated with electric shocks (fear conditioned), cause hypoalgesia in a finger withdrawal test following thermal stimulation [93]. In contrast, anxiety caused by electric shock induces hyperalgesia [93]. Although the direct role of the amygdala in these effects has not yet been shown in humans, the results in rodents are consistent with clearly defined functions of the amygdala in emotional responses to different aversive situations [2].

The amygdala thus can participate in adaptive processes leading to alleviation of pain sensation but its role is not limited to antinociception. While CeA lesion can block the antinociception induced in the tail-flick test by electrical shock pre-exposure, the affective hyperalgesia, measured by the latency to vocalize after the shock is also reduced by the same CeA lesion [92]. Interestingly, similar results are observed following lesion of the BSTL [92]. Pharmacological manipulation of the CeA can also increase visceromotor and/or somatomotor reflexes in naïve animals, as shown by intra-CeA injection of agonists to group I mGluRs [76, 77]. Similarly, CGRP and CRF administration in the CeA can induce mechanical hypersensitivity [82, 84], which is in contradiction with older studies reporting antinociceptive effects of these peptides [58, 59] (see below). Finally, corticosterone implants in the CeA sensitize the visceromotor reflexes to colorectal and urinary bladder distension [87, 88] and increases mechanical sensitivity [89]. The amygdala is thus likely to contribute to both analgesia and hyperalgesia.

### Amygdala, sustained pain, hyperalgesia and affective component

Persistent pain, such as inflammatory or neuropathic pain, has a different profile than acute pain. While animal studies often don’t address all the symptoms described in humans, the models that are used can display spontaneous nociceptive behaviors, hyperalgesia and/or allodynia, aversion as well as the emotional consequences of pain such as anxiety and depression [94].

The role of the CeA in sustained pain has been examined after intraplantar injection of formalin (somatic inflammatory pain), intraperitoneal injection of acetic acid (visceral inflammatory pain), intraarticular injection of kaolin and carrageenan (somatic inflammatory pain), and ligation/compression of the sciatic nerve (neuropathic pain) (Table 2). Generally, the manipulation of CeA activity, either by activation or by inactivation, did not modify the spontaneous nociceptive behaviors [30, 71, 72]. However, persistent pain leads to increased neuronal activity and synaptic transmission in the CeA (Table 1) which may be involved in the induction and/or maintenance of hypersensitivity observed in these models [5, 6, 33, 38]. Thus, inhibiting the extracellular signal-regulated kinase (ERK) activation in the CeA decreases mechanical, but not thermal, hyperalgesia in formalin and arthritis models, while the direct activation of ERK in the CeA is sufficient to produce mechanical hyperalgesia in naïve animals [30, 91]. In addition, intra-CeA injections of mGluRs antagonist (groups I and III) or of CGRP1 or CRF1 antagonists alleviate the increased withdrawal reflexes as well as audible and ultrasonic vocalizations in monoarthritic animals [78, 79, 83, 85, 86]. In neuropathic rats, activation of GABA-A receptors in the CeA also diminishes mechanical hypersensitivity [74]. These data suggest that changes in the activity and neurochemistry of the CeA contribute to the exacerbation of nociceptive responses in persistent pain.

The affective and emotional dimensions of pain can also be under the influence of the amygdala. The effects of pharmacological manipulations of the CeA on vocalizations reported above (see also Table 1) are pertinent in this context. Indeed, while audible vocalizations induced by a nociceptive stimulus are believed to reflect nocifensive responses, ultrasonic vocalizations would reflect an affective pain response [78, 95]. A widely used test to assess the affective/emotional component of pain, is the pain-induced conditioned place aversion. This behavior is suppressed in the intraplantar formalin and intraperitoneal acetic acid models by bilateral lesions of the CeA [72, 73] or by injection of β-adrenoceptor antagonist [81]. Similarly, intra-CeA administration of a GABA-A receptor agonist, a NMDA antagonist or a group I mGluR antagonist reduces the place avoidance behavior in neuropathic rats [74, 75].

Finally, the CeA may be a part of the mechanism linking sustained pain to anxiety and depression-like states. It has been shown that the models of monoarthritic pain or of sciatic nerve ligation induce anxiety that is correlated with the activation of the CRF system in the CeA. The expression of this neuropeptide, which has a well established role in anxiety, is increased in the CeA of neuropathic animals [39, 40], while the intra-CeA injection of CRF1 antagonist reduces anxious behaviors produced by knee monoarthritis [85, 86]. In addition, the pronociceptive effects of corticosterone implants in the CeA are accompanied by anxiety-like behaviors [90].

In humans, brain imaging studies [1] demonstrated changes in the activity of the amygdala of patients with irritable bowel syndrome [45], arthritis [96] or mononeuropathy [97], suggesting that the amygdala may be involved in the emotional aspects related to these pathologies. Interestingly, a brain imaging study showed the participation of an amygdala - anterior cingulate cortex circuit in the higher subjective perception of pain in healthy subjects experiencing sadness [98]. Conversely, viewing pictures of a romantic partner reduced self-reported pain, in association with activation of the amygdala [99]. These data reinforce the role of amygdala in emotional impact on pain.

In summary, the amygdala, particularly the CeA, may have a mainly antinociceptive influence in acute/phasic pain conditions associated with situations of stress or fear, and a mainly pronociceptive influence in persistent/tonic pain conditions which concerns the sensory as well as the emotional and affective dimensions.

### Potential mechanisms with several actors

Some information is available on the cellular and molecular mechanisms by which the amygdala can exert its bidirectional effects on pain parameters. Projections from the PB to the CeA give direct information about the perceived nociceptive stimulation through glutamatergic and peptidergic synapses [100–102], while the glutamatergic projections from the BLA to the CeA can bring polymodal information with an affective valence [5]. This latter input can be turned into an inhibitory control through the intercalated cell masses, functionally placed between the BLA and the CeA [4, 12]. The influence of the CeA on its targets will depend on the confrontation of these inputs with other afferent information. The internal network of the CeA and of the central extended amygdala is based on GABAergic inhibitory neurons [103], and the output of this system can either be inhibitory or disinhibitory on target structures [4, 104, 105]. Changes in the level of pain perception seem to involve the projection of the CeA to the centers of descending pain control, especially the PAG and the ventromedial reticular formation [47], while the influence of the amygdala on the emotional and affective dimensions of pain arises from a larger network that involves indirect connections with cortical regions such as the insular and the cingulate cortex [5, 20].

It is now clearly established that persistent pain causes long-lasting changes in the activity of the CeA that could account for its pronociceptive influence on sensory and affective components of pain [5, 6]. In arthritic, visceral and neuropathic pain models, subpopulations of nociceptive neurons of the CeA exhibit increased membrane excitability, leading to higher spontaneous activity, as well as potentiation of synaptic transmission [27, 33, 35, 37, 38]. This synaptic plasticity, observed at both the PB-CeA and the BLA-CeA synapses can potentiate nociceptive transmission. Some specificity can be observed concerning pain-related plasticity in the CeA. Indeed, in arthritis model, the population of multireceptive neurons, responding to innocuous stimuli but preferentially to nociceptive stimuli, presents increased basal activity and increased responses to mechanical but not thermal nociceptive stimuli. A second neuronal population, normally insensitive to somatosensory stimulation, develops responses to mechanical, but not thermal nociceptive stimuli. In contrast, arthritis modifies neither the basal activity nor the responses of the nociceptive-specific neurons [33]. In addition, while the PB and the BLA can synapse onto the same neurons, but on different dendritic compartments [106], specific plastic changes can occurs with different pain models. While arthritic and neuropathic pain models potentiates both the PB-CeA and the BLA-CeA synapses [33, 38], a model of visceral pain only potentiates the PB-CeA synapses [27].

An intriguing observation is that the right CeA appears largely more implicated in persistent pain than the left CeA. Indeed, the activation of ERK after intraplantar formalin is restricted to the right CeA, and the blockade of ERK activation in the right CeA decreased mechanical hyperalgesia at both hindpaws, irrespective to the side of formalin injection [30, 31]. In the arthritis model, the enhanced background activity and evoked responses are observed only in the right CeA, as well as the decreased activity following injection of a PKA inhibitor [34]. Finally, in neuropathic rats, spontaneous activity and evoked responses increased in the left CeA 2 and 6 days after sciatic nerve ligation, but declined afterward, whereas these electrophysiological parameters increased in the right CeA at 14 days post-ligation [37]. The different implication of left and right CeA in pain processes could partially account for the conflicting reports indicating antinociceptive [58, 59] and pronociceptive [82, 84] effects of CRF and CGRP injected into the CeA of naive rats. The anatomical and functional basis of this lateralization remains unclear, but it suggests a strong relation between right amygdala and affective/emotional component of persistent pain.

**Figure 3:**
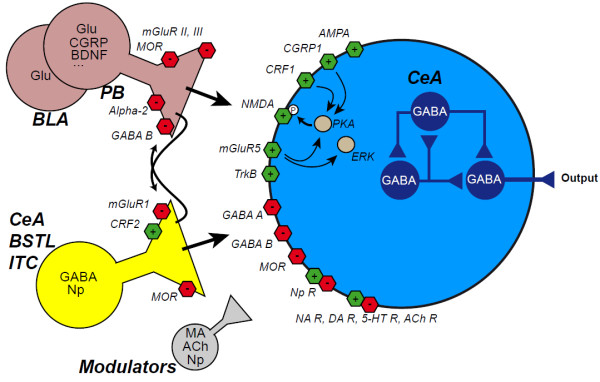
**Molecular actors in nociceptive processing in the CeA during arthritic pain.** The CeA received nociceptive inputs from glutamatergic (Glu) neurons in the basolateral amygdala (BLA) and in the parabrachial nucleus (PB). GABAergic innervation originates from intrinsic source (CeA and BSTL) and from the intercalated cell masses (ITC). Additional modulation is provided by monoaminergic (MA) and cholinergic (ACh) afferents and by neuropeptides (Np), especially from CeA, BSTL and PB. These neuromediators act on excitatory (green) and inhibitory (red) receptors at pre and post-synaptic levels. In the knee monoarthritis model [5], several mechanisms contribute to a enhanced neurotransmission. At pre-synaptic level, mGluR1 receptors inhibit GABA release, while mGluR from groups II and III decrease glutamate release. At post-synaptic level, mGluR5 receptors are involved in normal transmission in naive animals and NMDA and AMPA ionotropic receptors have also an enhanced activity increasing neuronal sensitivity. CGRP, from PB, and CRF, possibly from the central extended amygdala itself, activate their post-synaptic receptors, CGRP1 and CRF1, respectively. These receptors can activate the protein kinase A (PKA) and thus increase NMDA phosphorylation. The extracellular regulated-signal kinase ERK also contributes to the sensitization of CeA neurons, possibly through a cascade initiated by mGluR5 receptors. CRF can also decrease the activity of CeA neurons, via pre-synaptic CRF2 receptors increasing GABA release. This leads to the inhibition of CeA neurons by activation of post-synaptic GABA A and GABA B receptors and by inhibition of glutamate release by pre-synaptic GABA B receptors. Other neurotransmission system involving noradrenergic (NA, especially through pre-synaptic α_2_-receptors), dopaminergic (DA), serotonergic (5-HT) and cholinergic (ACh) receptors can modulate CeA activity, as well as other neuropeptides, among them opioids acting on pre- and post-synaptic mu receptors (MOR), and BDNF acting on TrkB receptors. Overall, these mechanisms lead to the modulation of the intra-CeA circuitry, based on GABAergic interactions (in blue) and of the CeA output.

A number of neurochemical and molecular mechanisms involved in the sensitization of the CeA have been elucidated. They especially implicate the modification of pre-and post-synaptic glutamatergic transmission, with an impact on GABAergic transmission, along with neuropeptidergic modulation [5, 6] (Figure 3). Following induction of experimental arthritis, the pre-synaptic expression of mGluR1 (group I) is increased, which in turn potentiates the synaptic transmission [35, 107]. This effect seems to involve a decreased inhibition, resulting in disinhibition of CeLC neurons and of glutamatergic terminals [108]. On the other hand, the activation of group II and III pre-synaptic mGluRs diminishes synaptic transmission [109–111]. At post-synaptic level, activation of group I mGluR (mGluR5) in the CeLC increased the excitatory response to nociceptive input in normal condition but not in arthritic condition [108], possibly involving PKA and ERK activation through reactive oxygen species [112]. In the same inflammatory pain model, NMDA ionotropic receptors are implicated in post-synaptic a sensitization, via a PKA-dependant mechanism [36, 113], while a NMDA-independent synaptic plasticity is induced in a neuropathic pain model [38]. Last, neuropeptide receptors (CRF1, CGRP1) contribute to this plasticity by favoring NMDA receptor phosphorylation through the recruitment of PKA and ERK kinases [82–84]. Together, these mechanisms lead to both increased nociceptive transmission and neuron sensitization in the CeA. The implication of CRF is here important as it also plays a role in stress response and anxiety. Moreover, while CGRP and CRF in CeA have a pronociceptive influence in arthritis model, the same neuropeptides can be acutely antinociceptive when directly injected in the CeA of control animals [58, 59]. This dual action, which likely implies qualitative and quantitative receptor changes, may also exist for other neuropeptides present in CeA, such as oxytocin or neurotensin. Opioid system in the CeA may be important in these phenomenons, but its contributions still remain elusive. The three types of opioid receptors (mu, delta, kappa) are expressed in the CeA, both pre- and post-synaptically, as well as their endogenous ligands [15, 16, 114–116]. The opioid system is a key actor in the CeA, due to the enkephalin contribution to intrinsic communications within the central extended amygdala and due to the contribution of the other opioid peptides to extrinsic CeA outputs [11]. The influence of the opioid system in the amygdala on pain is supported by various data, including the antinociceptive action of morphine in the CeA, the synergy between the CeA and the PAG co-delivery of opioid ligands [47] and the changes in CeA opioid receptor binding in neuropathic mice [117]. However, the circuit architecture underlying these actions, and the individual role of each peptide and receptor, is not yet clearly defined. For example, the mu-opioid receptors are pre-synaptically present in the CeA, where they can inhibit glutamate and GABA release from afferents [115, 118], but they also act post-synaptically to inhibit CeA interneurons and projection neurons [116] (Figure 3). Thus, these receptors can either inhibit or activate each component of the CeA. It is also not yet known how the opioid system interacts with other neurotransmission systems within the CeA, but several studies suggest that the CeA opioid system act downstream to agents that promote antinociception such as galanin or BDNF [60, 68]. Finally, adrenoceptors modulate pain processes in the CeA [64, 80, 81], especially through α_2_ adrenoceptors located on PB-CeA pre-synaptic elements which strongly regulate glutamate release [100].

### Beyond the central amygdala

While we focused this review on the CeA, it is necessary to remind the contribution of BSTL and BLA to pain process.

The CeA belongs to the central extended amygdala; it is largely interconnected with the BSTL and shares similar afferents, including polymodal and nociceptive inputs from the BLA and the PB [8, 21]. While there is no report of an influence of the BSTL on nociceptive sensitivity, a few studies addressed its role in pain-induced conditioned place aversion. Bilateral lesion of the BSTL [119], as well as intra-BSTL injection of a β-adrenoceptor antagonist [120, 121], α_2_-adrenoceptor agonist [122] or CRF1 antagonist [123] decrease place aversion in the intraplantar formalin and/or intraperitoneal acetic acid models. Moreover, noradrenaline and CRF release in the BSTL is enhanced in these pain models [120–122] and CRF mRNA is upregulated in the BSTL of neuropathic rats [40]. These data suggest that the BSTL can be involved in affective/emotional component of pain, maybe in a complementary manner with the CeA, especially in view of BSTL implication in anxiety [3].

The BLA, beyond its role as a mere input provider to the CeA and BSTL, also appears as an important actor in pain processes. Some observations suggest that the BLA and the CeA can have a parallel role in nocifensive responses. Stress- and fear-induced analgesia are reduced by intra-BLA infusion of cannabinoid CB1 receptors antagonists, of muscimol or of diazepam [124–127]; mu opioid agonist, including morphine, injected in the BLA decreases vocalizations to tail shocks [128] and thermal nociceptive sensitivity [129, 130]. Sustained pain also impacts the BLA as intraplantar formalin induces *c-fos* mRNA [24], arthritis increases the expression of the pro-nociceptive cytokine tumor necrosis factor α (TNF-α) [131] and neuropathy increases cell proliferation in the BLA [41]. A functional plasticity of BLA neurons is also observed in arthritis model, characterized by an increased in spontaneous and evoked activity and enhanced synaptic transmission. This pain-induced plasticity can be reversed by CRF1 antagonism [132].

The affective component of pain can also be modulated by the BLA. The formalin-induced conditioned place aversion is reduced by lesion of the BLA [72], as it is the case for the CeA and the BSTL, but also by BLA injection of NMDA antagonist or of morphine [133]. In addition, neutralizing BLA TNF-α with Infliximab antibodies reduced anxiety-like behaviors associated to arthritis [131]. Finally, CRF1 antagonism in BLA of arthritic rats decreased mechanical hypersensitivity, but also pain-related vocalizations and anxiety [132].

These influences of BLA on sensory and affective/emotional aspects of pain can, at least in part, involve its outputs to the CeA and/or BSTL. However, the BLA also appears to be implicated in pain-related cognitive impairment. Indeed, in arthritic rats, blockade of pain-induced plasticity in the BLA with CRF1 antagonism reversed the deficit in decision making evaluated in a gambling task. This deficit has been shown to be dependent upon a deactivation of the medial prefrontal cortex driven by the hyperactivity in the BLA. Importantly, this mechanism appears independent of the CeA [132]. Thus, the connections of the BLA with the cerebral cortex, especially prefrontal, cingulate and insular cortices, could allow the amygdala to influence both the affective/emotional and the cognitive aspects of pain. In addition, the dense BLA input to the ventral striatum [9, 10] could also participates in pain-induced changes in motivation and goal-directed behaviors.

## Conclusions

The data from the literature show that the amygdala, and in particular the CeA, contributes to pain processes. Its anatomical and functional relations with pain ascending systems, with pain facilitating or inhibitory descending systems, and with affective and cognitive centers are placing the CeA in a critical position within the pain matrix. Acute pain reactions only require CeA if they occur in specific emotional or adaptive contexts. On the other side, persistent and chronic pains alter CeA activity, which influences the pain experience and the related emotional, affective and motivational states. Reciprocally, these states can modify the amygdala processing of information and its impact on pain.

The amygdala has been mostly considered for its roles in emotion, especially in fear [2, 4]. While most studies focused on the BLA, recent researches on the fear circuit disclosed a part of the CeA microcircuitry involved in fear conditioning [104, 105]. It is very likely that a similar, if not the same, circuit underlies CeA functions in pain. Indeed, a recent study showed for the first time that the “nociceptive” amygdala (CeLC) undergoes synaptic plasticity at the PB-CeA and the BLA-CeA synapses during fear conditioning [134]. This result emphasizes the need to consider the strong relation between pain and emotion. The contribution to the pain matrix of the CeA, of other components of the amygdala and of the central extended amygdala, should foster researches in the context of chronic pain and the associated anxio-depressive disorders.

## Abbreviations

BLA: Basolateral amygdala
CeA: Central amygdaloid nucleus
CeL: Lateral part of the central amygdaloid nucleus
CeLC: Capsular part of the central amygdaloid nucleus
CeM: Medial part of the central amygdaloid nucleus
CGRP: Calcitonin gene-related peptide
CRF: Corticotropin-releasing factor
ERK: Extracellular signal-regulated kinase
mGluR: Metabotropic glutamatergic receptor
PB: Parabrachial nucleus
PKA: Protein kinase A
TNF-α: Tumor necrosis factor α.
